# COVID‐19 vaccine‐induced Radiation Recall Dermatitis: Report of a case

**DOI:** 10.1002/ccr3.5490

**Published:** 2022-02-23

**Authors:** Mozhdeh Sepaskhah, Fatemeh Ansari Asl, Mehrnoosh Taheri, Mojgan Akbarzadeh Jahromi

**Affiliations:** ^1^ Department of Dermatology School of Medicine Shiraz University of Medical Sciences Shiraz Iran; ^2^ Molecular Dermatology Research Center Shiraz University of Medical Sciences Shiraz Iran; ^3^ Department of Pathology School of Medicine Shiraz University of Medical Sciences Shiraz Iran

**Keywords:** COVID‐19 vaccines, mast cells, radiation recall dermatitis, radiodermatitis

## Abstract

Radiation Recall Dermatitis (RRD) is an inflammatory process in the site of irradiation, induced by physical and medical agents. Few cases of RRD in the skin and lung have been reported after COVID‐19 vaccination. Here, we report radiation recall dermatitis after both doses of inactivated SARS‐CoV‐2 vaccine (Sinopharm, China).

## Graphical abstract

Radiation recall dermatitis should be considered among the differential diagnoses of skin eruptions, in the appropriate clinical settings, after several kinds of COVID‐19 vaccinations, including inactivated SARS‐CoV‐2 vaccine (Sinopharm, China).

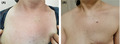

## 1 INTRODUCTION

Radiation recall dermatitis (RRD) is an inflammatory process in the site of irradiation, induced by physical and medical agents. Few cases of RRD in the skin and lung have been reported after COVID‐19 vaccination. Here, we report radiation recall dermatitis after both doses of inactivated SARS‐CoV‐2 vaccine (Sinopharm, China).

Radiation recall dermatitis (RRD) is an inflammatory disorder triggered by some medications and physical agents at the site of previous irradiation. This reaction is reported in the skin and other organs, like lung, muscle, and intestine, and is mainly precipitated by anti‐cancer medications; however, other drugs can also induce RRD (e.g., some antibiotics, statins, letrozole, and tamoxifen). ^1^, ^2^

Recently, a few cases of RRD have been reported after different types of COVID‐19 vaccine in the skin and lung.^3^, ^4^, ^5^

Here, we report a case of radiation recall dermatitis after inactivated SARS‐CoV‐2 vaccine (Sinopharm, China), repeated after rechallenge in the second dose of vaccine.

## 2 CASE REPORT

A 50‐year‐old woman presented to our outpatient dermatology clinic with an erythematous, pruritic skin rash over the upper chest and neck one month before referral. The lesions started one week after the second dose of the SARS‐CoV‐2 vaccine (Sinopharm, China). Similar lesions (less extensive and less symptomatic) had developed after the first dose of vaccine, injected three weeks before the second dose.

The patient is a known case of breast cancer (invasive ductal carcinoma) of the left breast that had undergone radical mastectomy and post‐operation chemotherapy and radiotherapy 15 years ago. Since then, she has had a routine follow‐up without any relapse of breast cancer.

**FIGURE 1:**
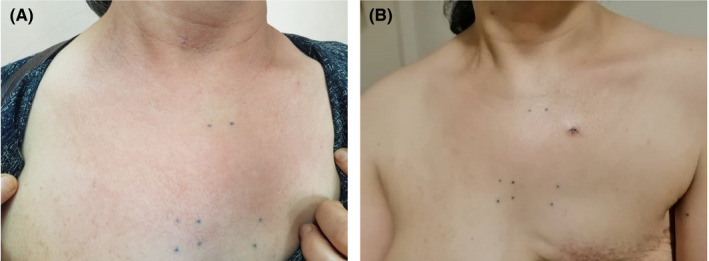
Clinical features. (A) Erythematous, ill‐defined plaques on the upper chest and neck (previous radiotherapy site), with focal dry desquamation (B) Significant improvement of the lesions after 10 days of treatment

**FIGURE 2:**
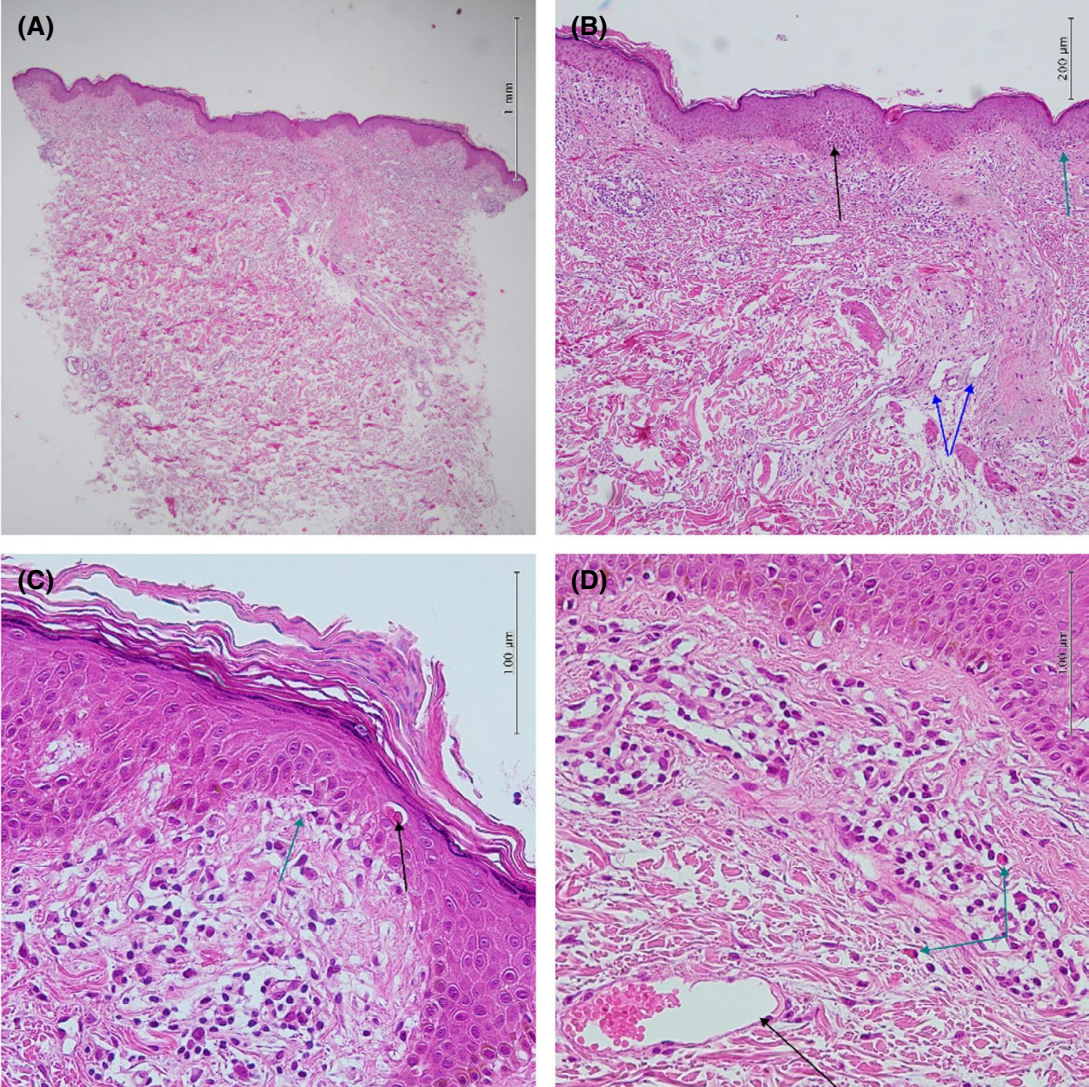
Histopathological features. (A) Hyperkeratosis, focal spongiosis, and upper dermal perivascular infiltration [Hematoxylin and eosin (H&E) stain, 40×] (B) Hyperkeratosis, parakeratosis, focal spongiosis (black arrow), focal basal cell degeneration (green arrow), and dilated vessels in the dermis (blue arrows) (H&E stain, 100×). (C) Basal cell degeneration (green arrow) and high apoptotic cells (black arrow) in the epidermis (H&E stain, 400×). (D) Superficial dermal perivascular, and mononuclear cell infiltrate admixed with eosinophils (green arrows) and dilated blood vessels (black arrow) (H&E stain, 400×)

**FIGURE 3:**
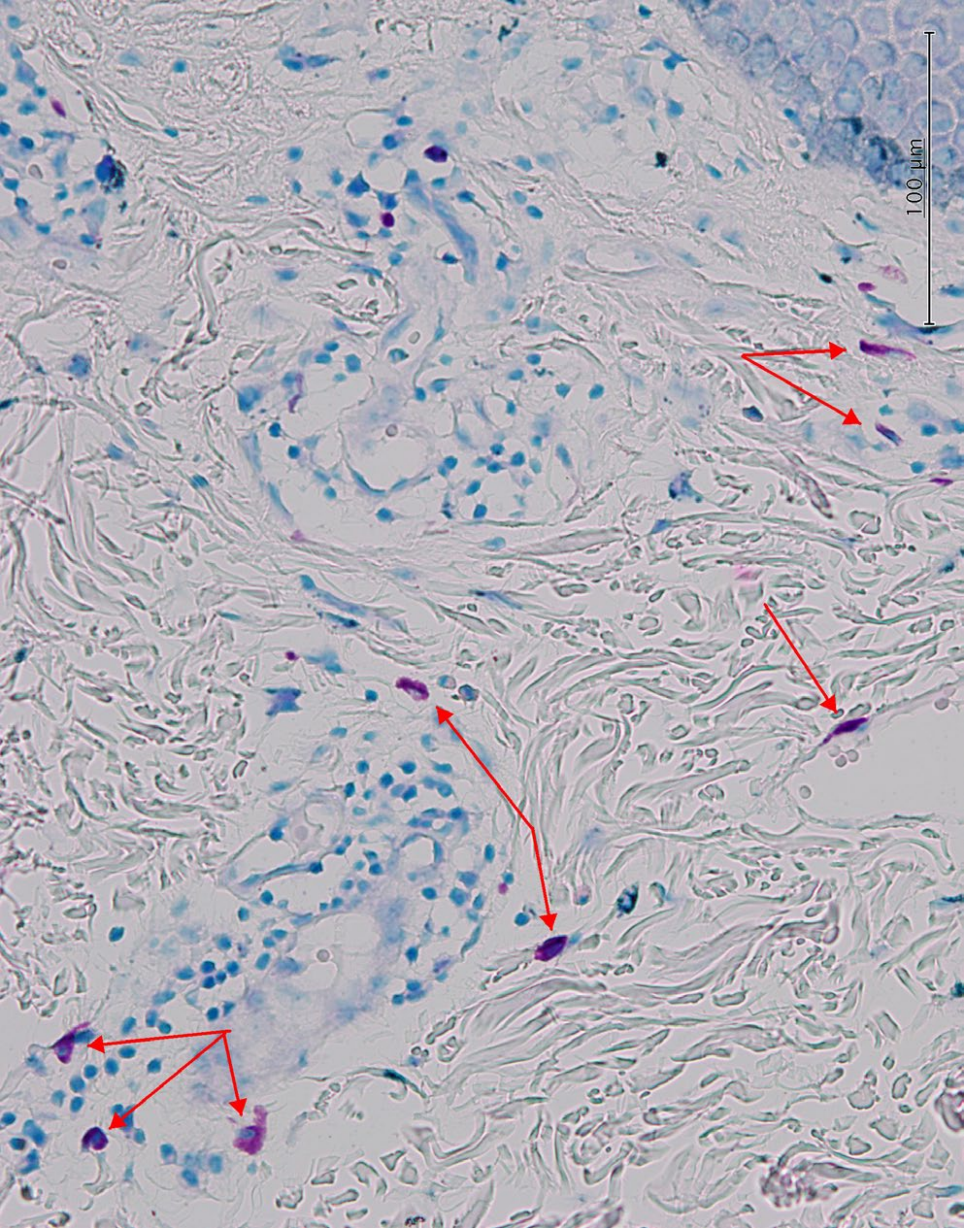
Histopathological features. Increased mast cell infiltration (red arrows) in the upper dermal infiltration (Giemsa stain, 400×)

On examination of the skin, relatively ill‐defined erythematous plaque with focal scaling and mild lichenification was observed on the upper chest, on the irradiation window area marked by tattoo (Figure 1A). A skin punch biopsy was performed, and the histopathological examination revealed hyperkeratosis, parakeratosis, mild irregular acanthosis, mild spongiosis, focal basal cell degeneration with few Civatte bodies, and high apoptotic cells. The upper dermis was infiltrated by perivascular lymphocytes and eosinophils and contained dilated blood vessels (Figure 2A‐D). Some mast cells were also visible in the upper dermis (Figure 3).

The differential diagnosis included erysipelas, carcinoma erysipeloides, and contact dermatitis. Erysipelas and carcinoma erysipeloides were excluded by lack of characteristic histological features. Contact dermatitis was a less relevant clinical diagnosis, due to the recurrence of rash at the previous radiotherapy site, and absence of recent exposure to any topical medication at the site of dermatitis or new systemic agent or medication.

According to the clinical and histopathological features, diagnosis of radiation recall dermatitis was made, and she was treated with oral prednisolone (15 mg daily) for three days, once‐daily application of mometasone furoate ointment, and frequent daily application of emollient. The signs and symptoms improved in a 10‐day follow‐up (Figure 1B).

The patient experienced no dermatitis after injection of the third dose of COVID‐19 vaccine, without pre‐ or post‐medication with steroids.

The patient signed written informed consent to permit the publication of the case report without identifying data and to use the photography for publication. The researchers committed to maintaining the patient confidentiality. Institutional ethics committee approved the case report (ethics code: IR.sums.med.rec.1400.325).

## 3 DISCUSSION

The radiation recall dermatitis represents an inflammatory process triggered by different agents in the previously irradiated body area. The most common triggering agents are drugs, especially anti‐tumor ones. However, other medications have also been discussed as causes of RRD, including antibiotics,^1^ simvastatin,^6^ letrozole,^7^ and tamoxifen.^8^ The inflammation can involve not only the skin but also the lung, esophagus, small intestine, and some other organs.^2^

The radiation recall dermatitis had not been reported after any vaccination before the COVID‐19 vaccination was started. Few reports of RRD after COVID‐19 vaccination have been recently published.^3^, ^4^, ^5^ The radiation recall reactions occurred after the adenovirus vector vaccine (Oxford‐AstraZeneca, UK),^5^ inactivated virus vaccine (Sinovac Coronavac, China),^3^ and RNA virus vaccine (Moderna, USA).^4^ Our case developed radiation recall dermatitis after injection of an inactivated virus vaccine (Sinopharm, China).

The previous cases of radiation recall dermatitis after COVID‐19 vaccination presented with well‐demarcated, erythematous, and indurated plaques^3^ and lesions with marked erythema and dry desquamation.^5^

Although only some of the previous cases of radiation recall dermatitis have undergone skin biopsy, some common features are described in the reported cases, including vacuolar changes in the dermo‐epidermal junction, apoptotic epidermal cells, vascular dilatation, and dermal infiltration of lymphocytes and eosinophils.^2^, ^9^ The same features were observed in the histopathological examination of our patient.

Ristić hypothesized the role of mast cells in the pathogenesis of RRD.^10^ Increased number of mast cells in the dermal inflammatory infiltration of our case may favor this hypothesis.

The time lapse between radiation and RRD in our patient (15 years) was among the most prolonged periods presented in the literature,^2^ but the time between the insult and development of the rash (7 days) was in concordance with most of previous cases.^2^ The radiation recall rash developed from three hours to five days after COVID‐19 vaccine injection.^3^, ^4^, ^5^

The recurrence of RRD on re‐exposure with the causative agent is controversial.^1^ The reported cases of RRD after COVID‐19 vaccination developed the rash either only after the injection of the second dose of vaccine (and not the first dose) or after the first dose and before the injection of the second dose of vaccine^3^, ^4^, ^5^; so, the possibility of recurrence on rechallenge was absent or not assessable. Nevertheless, our patient experienced RRD after injection of the first and second vaccine doses (with a more severe reaction after the second dose), but she experienced no rash after a second rechallenge. Discontinuation of the precipitating agent of RRD has been recommended, but not insisted. Recurrence of RRD after rechallenge is not the rule. Lack of recurrence after rechallenge with causative agent has been reported in a noticeable number of RRD cases.^1^ So, the existing data do not support the prohibition of the next doses of vital agents like COVID‐19 vaccine after episodes of RRD.

The radiation recall dermatitis has been treated with oral and/or topical corticosteroids, antibiotics, emollients, and analgesics,^2^ and the rash in our patient improved by a short course of oral and topical steroids.

## 4 CONCLUSION

There may be an increasing incidence of RRD after the COVID‐19 vaccination. So, the physicians should be cautious about this diagnosis in patients previously treated with radiation and after the COVID‐19 vaccine injection to recognize and treat it promptly.

## CONFLICT OF INTEREST

The authors declare no conflict of interest.

## AUTHOR CONTRIBUTIONS

Mozhdeh Sepaskhah involved in clinical diagnosis of the case, treatment planning, writing the manuscript draft, and final version approval. Fatemeh Ansari Asl involved in data acquisition, patient follow‐up, critical review of the draft, and final version approval. Mehrnoosh Taheri involved in clinical diagnosis of the case, critical review of the draft, and final version approval. Mojgan Akbarzadeh Jahromi involved in pathological confirmation of the diagnosis, critical review of the draft, and final version approval.

## ETHICAL APPROVAL

The researchers committed to maintaining the patient confidentiality. Institutional ethics committee approved the case report (ethics code: IR.sums.med.rec.1400.325).

## CONSENT

The patient signed written informed consent to permit the publication of the case report without identifying data and to use the photography for publication. The researchers committed to maintaining the patient confidentiality. Institutional ethics committee approved the case report (ethics code: IR.sums.med.rec.1400.325).

## Data Availability

Data sharing is not applicable to this article as no new data were created or analyzed in this study.
